# Exosomes impact survival to radiation exposure in cell line models of nervous system cancer

**DOI:** 10.18632/oncotarget.26300

**Published:** 2018-11-16

**Authors:** Oliver D. Mrowczynski, Achuthamangalam B. Madhankumar, Jeffrey M. Sundstrom, Yuanjun Zhao, Yuka Imamura Kawasawa, Becky Slagle-Webb, Christine Mau, Russell A. Payne, Elias B. Rizk, Brad E. Zacharia, James R. Connor

**Affiliations:** ^1^ Department of Neurosurgery, Pennsylvania State University College of Medicine, Hershey, PA 17033, USA; ^2^ Department of Ophthalmology, Pennsylvania State University College of Medicine, Hershey, PA 17033, USA; ^3^ Department of Pharmacology, Biochemistry and Molecular Biology, Institute for Personalized Medicine, Pennsylvania State University College of Medicine, Hershey, PA 17033, USA

**Keywords:** exosomes, radiation, resistance, glioma, glioblastoma

## Abstract

Radiation is utilized in the therapy of more than 50% of cancer patients. Unfortunately, many malignancies become resistant to radiation over time. We investigated the hypothesis that one method of a cancer cell's ability to survive radiation occurs through cellular communication via exosomes. Exosomes are cell-derived vesicles containing DNA, RNA, and protein. Three properties were analyzed: 1) exosome function, 2) exosome profile and 3) exosome uptake/blockade. To analyze exosome function, we show radiation-derived exosomes increased proliferation and enabled recipient cancer cells to survive radiation *in vitro*. Furthermore, radiation-derived exosomes increased tumor burden and decreased survival in an *in vivo* model. To address the mechanism underlying the alterations by exosomes in recipient cells, we obtained a profile of radiation-derived exosomes that showed expression changes favoring a resistant/proliferative profile. Radiation-derived exosomes contain elevated oncogenic miR-889, oncogenic mRNAs, and proteins of the proteasome pathway, Notch, Jak-STAT, and cell cycle pathways. Radiation-derived exosomes contain decreased levels of tumor-suppressive miR-516, miR-365, and multiple tumor-suppressive mRNAs. Ingenuity pathway analysis revealed the most represented networks included cell cycle, growth/survival. Upregulation of DNM2 correlated with increased exosome uptake. To analyze the property of exosome blockade, heparin and simvastatin were used to inhibit uptake of exosomes in recipient cells resulting in inhibited induction of proliferation and cellular survival. Because these agents have shown some success as cancer therapies, our data suggest their mechanism of action could be limiting exosome communication between cells. The results of our study identify a novel exosome-based mechanism that may underlie a cancer cell's ability to survive radiation.

## INTRODUCTION

More than fifty percent of cancer patients, including patients with the most devastating central nervous system malignancy, glioblastoma [1, 2, 3], receive radiation as a critical component of their standard treatment regimen [4]. One reason for the dire prognosis of cancer is its ability to elude standard radiotherapy [5]. Glioblastoma is molecularly heterogeneous and this intratumoral heterogeneity and environmental modification traits of cancer are accentuated during treatment. Even in the face of surgical resection and adjuvant chemoradiation, recurrence and progression are nearly universal. While the inherent heterogeneity of aggressive cancers likely mediates part of therapeutic resistance, other factors may also play important roles. We propose that cellular communication via exosomes is critical to the ability of cancer cells to survive radiation therapy. Exosomes are nanometer-sized vesicles [6–9] released by cells that contain genetic components of the parent cancer cell from which they were derived [10–15], and have broad ranging effects on the tumor microenvironment [16, 17, 18]. A seminal paper by Skog et al and another by the Breakefield group have demonstrated that exosomes transfer their components to recipient cells in the body [19, 20]. Exosomes have a protective lipid bilayer and are small enough to permit travel throughout the body without being degraded [21, 22, 23, 24]. It has been demonstrated that stressors such as hypoxia can change exosomal content and functionally impact the local cell population [25, 26]. Recent studies have also demonstrated that ionizing radiation increases the release of exosomes from glioblastoma cells and alters their contents rendering the exosomes more oncogenic [27]. Although changes in exosome content due to radiation have been identified [27, 28, 29, 30], the potential role of exosomes in induction of radiation survival and proliferation in recipient cancer cells induced by these radiation-derived exosomes has not been explored.

To that end, we undertook interrogation of the impact of radiation on exosome profile and the effects of radiated exosomes on recipient cells in the surrounding tumor environment. We explore whether exosomes secreted by radiated cancer cells that are subsequently taken up by recipient cells render those recipient cells more apt to radiation therapy. This concept may be especially critical for inherently radiation resistant cancer cells, as well as cancer cells at the border of the radiation treatment field where sub-lethal doses of radiation may act as a stressor causing the release of oncogenic exosomes. Although highly controversial, a recent study by Duma et al [31] demonstrated that utilizing a technique coined “Leading edge radiation” and expanding the radiation treatment margins led to significantly better outcomes in glioblastoma patients when compared to standard radiation protocols.

Lastly, exosomes are taken up by a multitude of mechanisms, mediated by tetraspanin (CD81), proteoglycans, and/or lipid rafts. The use of antibodies to the CD81 protein on the cell surface of exosomes as well as using heparin, and simvastatin to block exosome uptake are just beginning to be investigated [32, 33, 34, 35, 36, 37]. Heparin and statins have also been suggested to have anti-tumor effects [38–42, 43, 44], but the underlying mechanism is unclear. Simvastatin is of particular interest for CNS malignancies due to its hydrophobicity and thus increased uptake into the brain through and intact blood brain barrier [43]. By interrogating these compounds in our model, we ultimately aim to advance understanding of existing treatments whose mechanism may be through exosome inhibition.

## RESULTS

### Characterization of exosome size and quantity released from radiated and non-radiated glioblastoma cells

**Figure 1:**
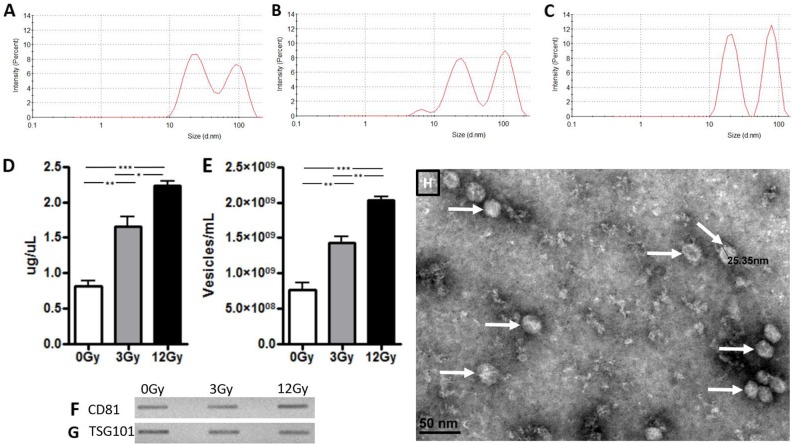
Exosome confirmation analysis in U87 glioma cells Zetasizer analysis in Panels A-C demonstrate that size of the exosomes was not affected by radiation exposure but there is a dose dependent increase in release intensity. **(A)** At 0Gy radiation, exosomes had a release intensity of 8.5 and 7.1. **(B)** At 3Gy radiation, the release intensity was 7.98 and 9.1. **(C)** At 12Gy radiation, the release intensity was 11.3 and 12.2. Exosomes were then quantified with **(D)** BCA assay and **(E)** nanoparticle tracking analysis. Panels F and G demonstrate exosome release from U87 glioma cells before and after exposure of radiation at 3Gy and 12Gy with immunoblots of exosome confirmation markers **(F)** CD81 and **(G)** TSG101. **(H)** Electron microscopy visualization of exosomes (white arrows) from U87 cells. (^*^p<.05, ^**^p<.01, ^***^p<.001)

Figure 1 shows the purification of exosomes between 20-200 nanometers with dynamic light scattering on the Zetasizer particle size analyzer (Malvern Nano ZS). Two populations of exosomes were released from the U87 glioma cells, with average sizes of 24 nm and 93 nm respectively (Figure 1A–1C). The data indicate there are no apparent changes in size distribution with regards to radiation treatment. However, we did note an increase in exosomal release following radiation treatment in a dose-dependent manner, as shown by the number of particles, or exosome release intensity (Figure 1A–1C). This increase in radiation-induced exosome release was also confirmed with BCA protein analysis as well as nanoparticle tracking analysis (Figure 1D, 1E). Both analyses showed a 2-fold increase in exosome secretion after exposure to 3Gy radiation and a 3-fold increase in exosome secretion after exposure to 12Gy compared to control. Common surface protein markers that are highly expressed on exosomes include the tetraspanin family of proteins, which include CD81 and TSG101. These markers are expressed by exosomes from glioma cell lines with and without radiation treatment (Figure 1F, 1G). Exosomes from glioma cells were visualized and confirmed with electron microscopy (Figure 1H).

### Functional impact of exosomes derived from radiated and non-radiated cancer cell lines *in vitro*

**Figure 2:**
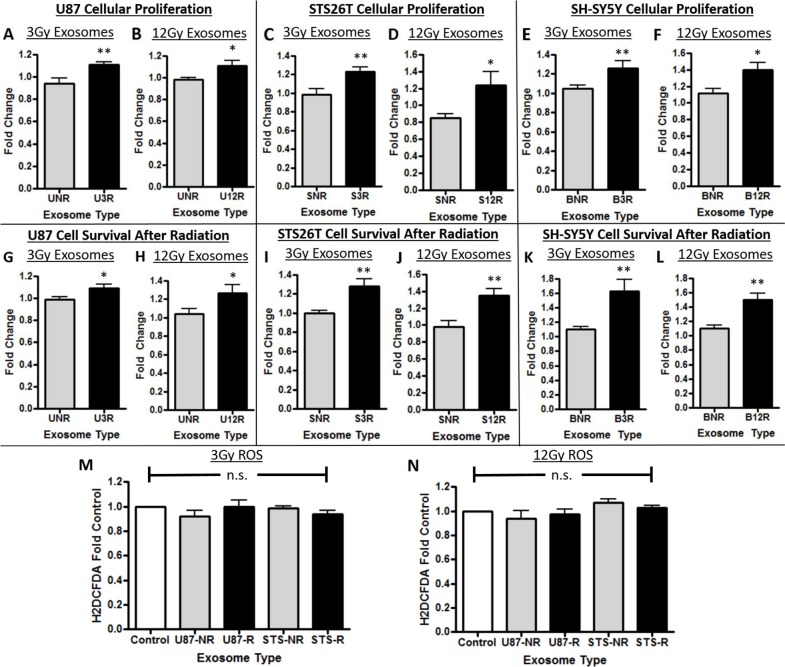
Cellular proliferation and survival effects of exosomes In panels A-F, U87 glioma cells are represented with a “U”, STS26T MPNST cells with an “S”, and SH-SY5Y neuroblastoma cells with a “B”. The number following the cell line letter is the dosage of radiation used; either 3R or 12R. The cells were either exposed to exosomes from not irradiated cells “NR” or to exosomes (Exos) from cells that received one of the two doses of radiation (“3R” or “12R”). The effect of the exosomes on cellular proliferation is shown in Panels A-F: **(A,B)** U87 glioma cells, **(C,D)** STS26T MPNST cells, **(E,F)** SH-SY5Y neuroblastoma cells. Increased recipient cancer cell survival after radiation due to the effect of exosomes in Panels G-L: **(G,H)** U87 glioma cells, **(I,J)** STS26T MPNST cells, **(K,L)** SH-SY5Y neuroblastoma cells) (^*^p<.05, ^**^p<.01). In Panels M and N are data showing there was no increase in reactive oxygen species of cells incubated with exosomes compared to control for either **(M)** 3Gy or **(N)** 12Gy radiated (represented with an “R”) or non-radiated (“NR”). The results show increased proliferation and increased survival to radiation when cells are exposed to exosomes from irradiated cells.

Naïve cancer cells incubated with exosomes purified from each of the cancer cell types radiated with 3Gy and 12Gy had a significant increase in cellular proliferation when compared to control (Figure 2A–2F). Furthermore, naïve cancer cells incubated with exosomes derived from radiated cancer cells had a significant increase in ability to survive radiation exposure when compared to control in all cell types (Figure 2G–2L). We then determined whether a decrease in production of reactive oxygen species (ROS) by these cell lines incubated with exosomes was a potential mechanism underlying the increase in cellular survival after radiotherapy. No changes in ROS production were found after radiation exposure by the cells incubated with and without exosomes (Figure 2M, 2N).

### Inhibition of enhanced cellular proliferation and survival *in vitro*

**Figure 3:**
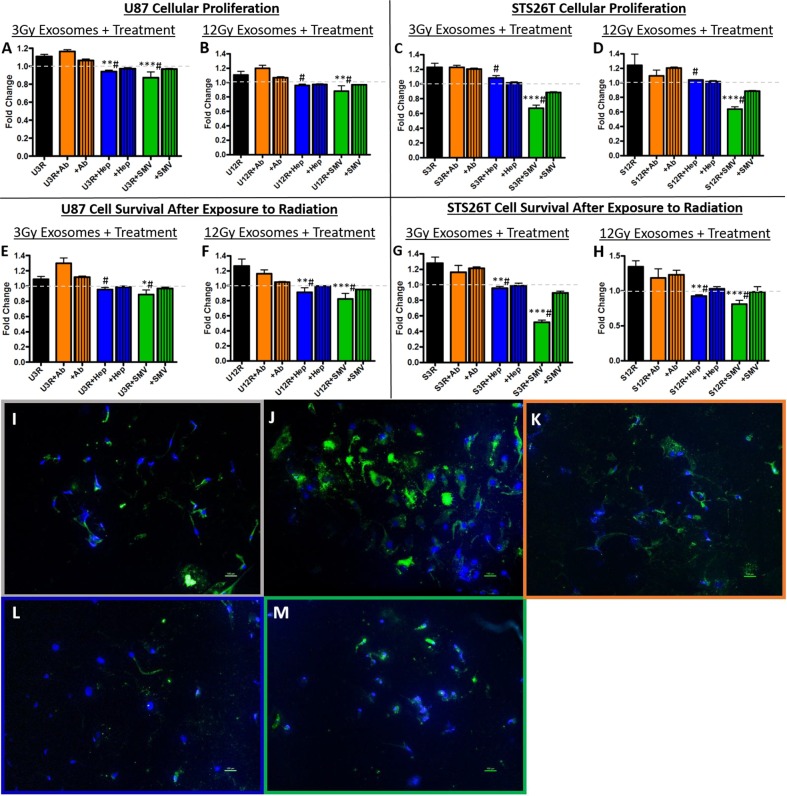
Exosome blockade analysis Panels A-D show heparin (Hep) and simvastatin (SMV) were able to decrease the proliferation induced by the radiation derived exosomes (rad exos) in **(A,B)** U87 and **(C,D)** STS26T cells. Panels E-H show Hep and SMV were able to decrease the cell survival conferred by radiation-derived exosomes in **(E,F)** U87 and **(G,H)** STS26T cells. The addition of CD81 antibody (+Ab) was not as effective on either proliferation or survival. Panels I-M show microscopic examination of uptake of exosomes labeled with green PKH67 fluorescence under the various conditions in U87 glioma cells. **(I)** Exosomes from non-radiation cells show minimal uptake whereas **(J)** exosomes derived from irradiated cells are taken up robustly. **(K)** Radiation-derived exosomes plus anti-CD81 antibodies had minimal effect on exosome uptake similar to exosomes from non-radiated cells. **(L)** Radiation-derived exosomes plus heparin. **(M)** Radiation-derived exosomes plus simvastatin. Hep (L) and SMV (M) both decreased uptake of fluorescently labeled radiation-derived exosomes when compared to fluorescently labeled radiation-derived exosomes without treatment (J). (^*^p<.05, ^**^p<.01, ^***^p<.001 significantly decreased compared to control, # previous increase due to radiation-derived exosomes is now not significant).

The addition of heparin or simvastatin blocked the oncogenic effects of the radiation-derived exosomes. Recipient cellular proliferation (Figure 3A–3D) and cellular survival after exposure to radiation (Figure 3E–3H) were both inhibited. The attempt to block exosome uptake with an antibody to the tetraspanin protein CD81 was not as effective as heparin or simvastatin, and thus heparin and simvastatin were chosen to move forward. To determine if the functional effects were due to decreased uptake of exosomes we performed microscopic analysis of exosome uptake. The radiation-derived exosomes (Figure 3J) are internalized more readily by recipient cells than their non-radiation derived counter parts (Figure 3I). Simvastatin and Heparin decreased the uptake of exosomes consistent with the effect of these compounds on the functional measures (Figure 3L, 3M).

### *In vivo* studies

**Figure 4:**
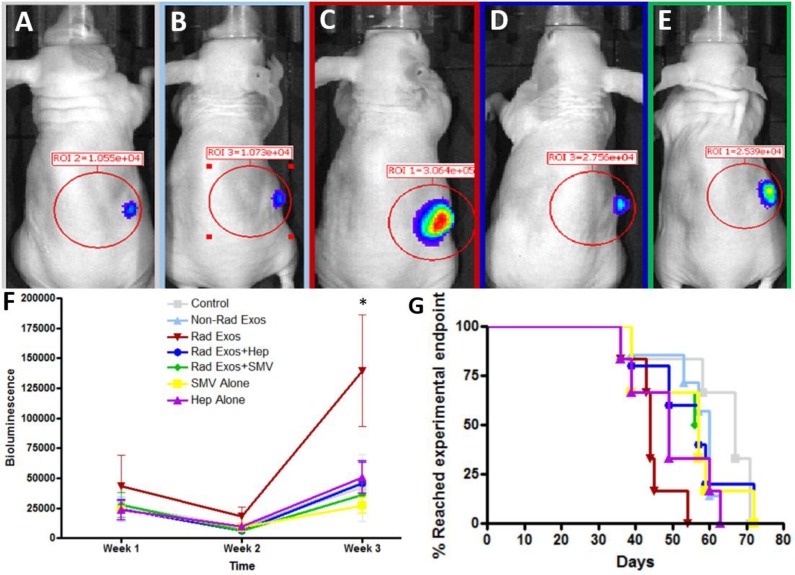
*In vivo* analysis of radiation derived exosome effect and therapeutic blockade Representative IVIS images of **(A)** Control **(B)** Non-radiation exosomes **(C)** Radiation-derived exosomes, **(D)** Radiation-derived exosomes plus daily heparin (Hep), **(E)** Radiation-derived exosomes plus daily simvastatin (SMV) treatment. Mice treated with radiation-derived exosomes had visually larger tumors when compared to control. When co-treating mice with radiation-derived exosomes plus heparin or simvastatin, the tumor size decreased and was comparable to control levels. **(F)** Tumor progression over time was quantified with IVIS counts. Mice treated with radiation-derived exosomes (represented as “Rad Exos”) had an increase in tumor progression and when co-treating with Hep or SMV tumor progression was similar to baseline (p<0.05). **(G)** Mice treated with radiation-derived exosomes had a decrease in survival time but when co-treating with heparin or simvastatin the mouse survival increased.

Representative images of the mice and their tumors are shown with IVIS (Figure 4A–4E). Though all seven groups started with similar average bioluminescent signals, there was enhanced tumor burden in the mice treated with radiation-derived exosomes (Figure 4F). This effect was abrogated with daily treatment of heparin or simvastatin (Figure 4F). Survival was consistent with the *in vivo* imaging results. Mice treated with radiation-derived exosomes showed a decrease in survival and co-treatment with heparin or simvastatin conferred a survival advantage (Figure 4G).

### Immunohistochemistry of tumor samples

**Figure 5:**
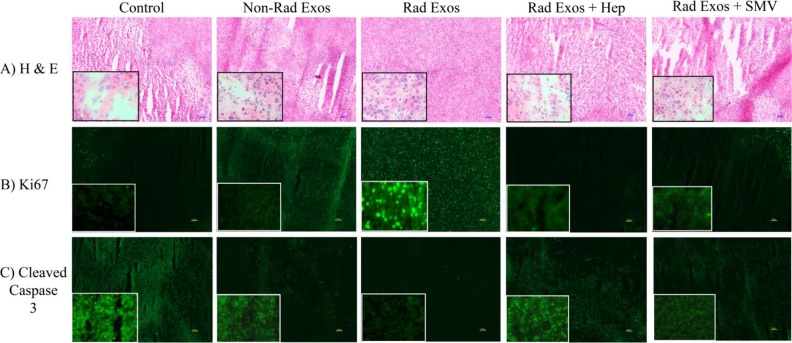
Immunohistochemistry of glioblastoma tumor samples from each group **(A)** H & E staining revealed increased necrotic tissue in the control saline treated tumors when compared to the radiation-derived exosome (Represented as “Rad Exos”) treated tumors. **(B)** Ki67 cellular proliferation marker analysis showed decreased proliferation in the control tumors when compared to the radiation-derived exosome treated tumors. **(C)** Cleaved caspase 3 marker for cell death increased in control tumors when compared to tumors treated with radiation derived exosomes. All of the effects associated with radiation-derived exosomes seen by immunohistochemical analysis were not present in tissue from tumors co-treated with heparin or simvastatin. The tumors from the heparin and simvastatin treated animals appeared similar to controls. The inserts are 40X images provided to show more cellular details within the tumors.

Immunohistochemical analysis of tumor tissue for markers of tumor growth, proliferation, and apoptosis was performed (Figure 5A–5C). H&E staining of tumor tissues showed increased amount of necrosis in the control saline treated tumors, when compared to tumors treated with radiation-derived exosomes. This phenotype reverted back to control with co-treatment of heparin or simvastatin (Figure 5A). Ki67 cellular proliferation marker analysis showed less proliferation in the control tumors compared to tumors treated with non-radiation and radiation-derived exosomes. The amount of Ki67 staining was similar to control in the tumors co-treated with radiation-derived exosomes and heparin or simvastatin (Figure 5B). Cleaved caspase 3 marker for cell death increased in control tumors, to a lesser extent in the tumors treated with non-radiation derived exosomes, and even less in the tumors treated with radiation-derived exosomes. (Figure 5C). Adding heparin and statin therapy to the tumors treated with the radiation-derived exosomes caused those tumors to have increased cell death (Figure 5C).

### Analysis of RNA and proteomic contents within exosomes

**Figure 6:**
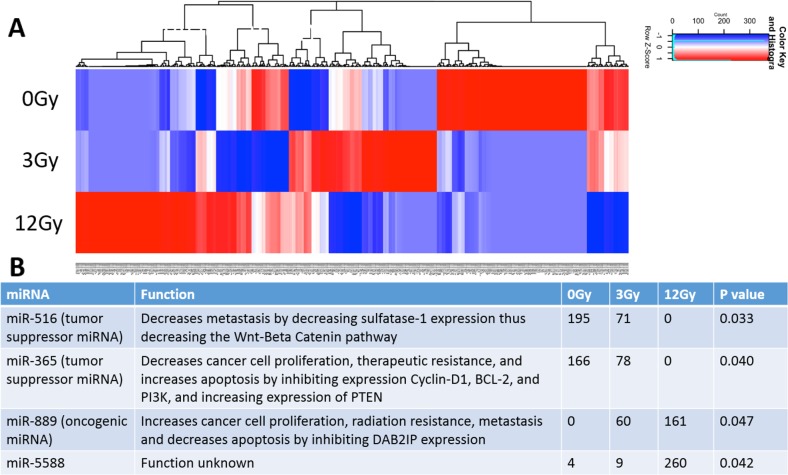
Analysis and comparison of miRNA contents within the non-radiation and radiation derived glioma exosomes **(A)** Distinct heat map profiles were generated for exosomes derived from cells exposed to 0Gy (control glioma exosomes), 3Gy (low radiation), and 12Gy (high radiation). A total of 516 miRNA were identified in the exosomes following irradiation **(B)** Table showing the 4 statistically significant exosomal miRNAs following irradiation. The oncogenic miRNAs and tumor suppressive miRNAs were up and down regulated, respectively.

A total of 516 miRNAs were found within the exosomes. Heat maps generated show differential miRNA profiles based upon the dose of radiation (Figure 6A). Figure 6B shows the 4 miRNAs that were identified as statistically significantly changed (p<0.05) and includes miR-516, miR-365, miR-889, and miR-5588. Moreover, it is noteworthy that the tumor suppressive miRNAs (miR-516 and miR-365) decrease when exposed to increasing radiation stress, while the oncogenic miR-889 increases when exposed to increasing radiation stress (Figure 6B).

**Figure 7:**
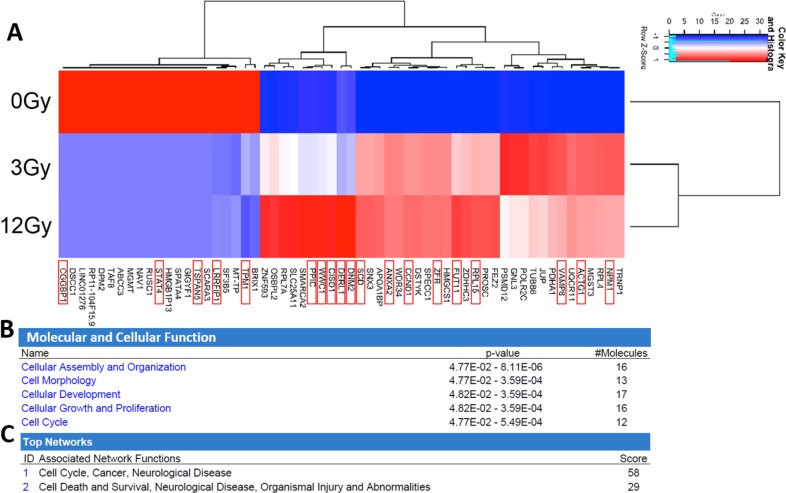
Analysis and comparison of mRNA contents within the non-radiation and radiation derived glioma exosomes **(A)** The change in expression levels of 59 mRNA were identified following irradiation (p<0.05). Distinct heat map profiles were generated for exosomes derived from cells exposed to 0Gy (control glioma exosomes), 3Gy (low radiation), and 12Gy (high radiation). mRNA that have been demonstrated to have oncogenic or tumor suppressive functionality are highlighted with a red box. There is clearly a dose response to the patterns of expression. Panels B-C: Ingenuity Pathway Analysis and comparison of mRNA contents within the non-radiation and radiation derived glioma exosomes. **(B)** Molecular and cellular function pathways most highly represented in the radiation derived exosomes **(C)** The mRNA networks most represented in the radiation derived exosomes.

The expression level of 59 mRNAs was altered in the exosomes in response to increasing radiation. Heat map profiles show differential expression based upon radiation dosage (Figure 7A). Oncogenic mRNAs significantly (p<0.05) upregulated following irradiation included Nucleophosmin 1 (NPM1), Actin Gamma 1 (ACTG1), Vesicle Associated Membrane Protein 8 (VAMP8), Ribosomal Protein L15 (RPL15), fucosyltransferase 11 (FUT11), Zinc Finger RNA Binding Protein (ZFR), Cyclin D1 (CCND1), Annexin A2 (ANXA2), Stearoyl-CoA desaturase (SCD), Dynamin 2 (DNM2), Derlin 1 (DERL1), mitoNEET (CISD1), Kibra (WWC1), and Peptidylprolyl Isomerase C (PPIC). Tumor-suppressive mRNAs found to be significantly downregulated following irradiation include Tropomyosin 1 (TPM1), LRR Binding FLII Interacting Protein 1 (LRRFIP1), Tetraspanin 5 (TSPAN5), Signal Transducer And Activator Of Transcription 4 (STAT4), CGG Triplet Repeat Binding Protein 1 (CGGBP1)[45–49]. The most highly represented molecular and cellular functional pathways in the radiation-derived exosomes include cellular assembly and organization, cell morphology, cellular development, cellular growth and proliferation, and cell cycle (Figure 7B). The most represented networks in the radiation-derived exosomes include cell cycle, cancer, cell death and survival, and organismal injury (Figure 7C).

**Figure 8:**
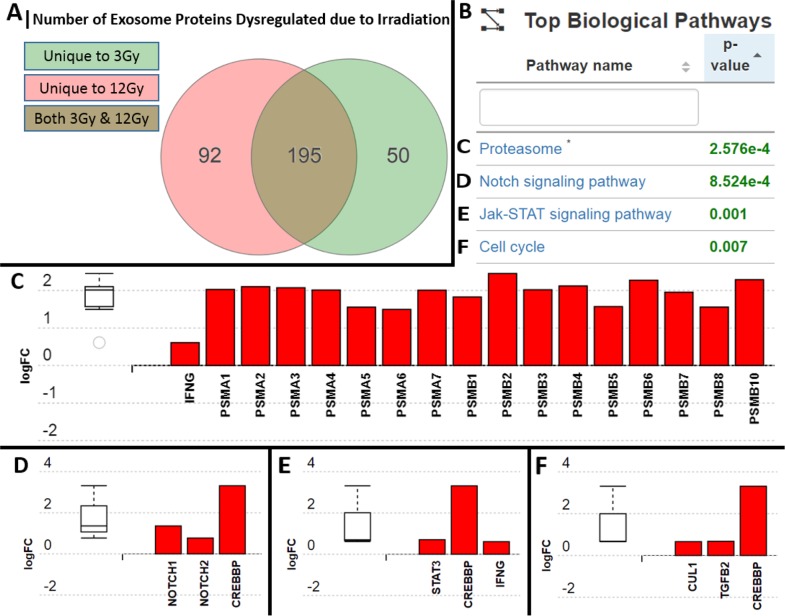
Analysis and comparison of protein contents within the non-radiation and radiation derived glioma exosomes **(A)** 50 proteins were unique to the 3Gy derived exosomes, 92 were unique to the 12Gy derived exosomes, and 195 were overlapping in both radiation dose derived exosomes in comparison to non-radiation derived exosomes **(B)** Table showing the 4 most statistically significantly represented protein profiles in the exosomes following radiation were **(C)** Proteasome pathway proteins **(D)** Notch signaling pathway proteins **(E)** Jak-STAT pathways proteins **(F)** Cell cycle pathway proteins. All of the proteins upregulated in the exosomes are known to be associated with increased resistance to radiation and increased cellular proliferation.

Over 1000 proteins were found within the glioma exosomes; 50 of which were unique to the 3Gy-derived exosomes, 92 which were unique to the 12Gy-derived exosomes, and 195 that were in both radiation dose-derived exosomes but not found in to non-radiation derived exosomes (Figure 8A). iPathwayGuide analysis revealed 4 significantly (p<0.05) represented biological pathways (Figure 8B) included the Proteasome pathway, the Notch signaling pathway, the Jak-STAT signaling pathway, and the cell cycle pathway (Figure 8C–8F). The expression of the proteasome pathway proteins within the radiation-derived exosomes were upregulated in comparison to their non-radiation derived counterparts (Figure 8C). iPathwayGuide analysis also revealed an upregulation of the oncoproteins STAT3, Notch1/2, Cullin1, Transforming Growth Factor-Beta 2 (TGF-B2), and cAMP-response element binding protein (CREBBP) (Figure 8D–8F).

## DISCUSSION

Exosomes are instrumental in a cancer cell's interaction with its microenvironment. In the present study, we explored whether the stress of radiation alters the dynamics of exosomes released from multiple cancer cell types. Three properties of the exosomes are analyzed: 1) function, 2) molecular and protein profile, and 3) uptake/blockade. We provide evidence that exposure to radiation treatment results in dose dependent increased secretion of exosomes and that these radiation derived exosomes have upregulated oncogenic and downregulated tumor-suppressive contents. We further show that these radiation-derived exosomes alter naïve recipient cancer cells *in vitro* and *in vivo* by increasing cellular proliferation, enabling cells to survive radiation exposure, and increasing tumor burden, and that these effects can be abrogated, in part, via blockade of exosome uptake with heparin and simvastatin. The functional impact of the exosomes on naïve recipient cells suggests there are alterations in exosome composition due to radiation. Thus, we assessed the changes in exosomal contents due to radiation.

We show that the radiation-derived exosomes have upregulation of oncogenic and downregulation of tumor suppressive miRNA, mRNA, and protein. Multiple RNA species changed as a result of radiation. Downregulation of miR-516, a tumor suppressive miRNA, decreases metastasis by decreasing sulfatase-1 expression leading to a decrease in the Wnt-Beta catenin pathway [50]. Downregulation of miR-365, a tumor suppressive miRNA, increases cancer cell proliferation, therapeutic resistance, and decreases apoptosis by disinhibiting expression of Cyclin-D1, BCL-2, and PI3K, while decreasing expression of PTEN [51–54]. Upregulation of miR-889, an oncogenic miRNA, increases cancer cell proliferation, radiation resistance, metastasis, and decreases apoptosis by inhibiting DAB2IP expression [55–57]. The upregulated oncogenic mRNA found have a functional influence on cancer cells by increasing cellular proliferation (ex. CCND1), radiation resistance (ex. WWC1), and exosome uptake (ex. DNM2)[58–71]. The downregulated tumor-suppressive mRNA found have a functional influence on cancer cells by decreasing cellular proliferation (ex. STAT4) and decreasing radiation resistance (ex. TPM1). Interestingly, previous studies have also shown that ANXA2, a prominent factor found in our study, to be major component within exosomes [72]. Some common glioblastoma exosome miRNAs previously discussed include miR-21, miR-451, miR-1, miR-320, and miR-574-3p [20],[9],[73]. Our 4 significant miRNAs are different which is likely due to our analysis focusing on the miRNA dysregulated due to radiation, rather than analyzing the most abundant miRNA found in the exosomes themselves.

**Figure 9:**
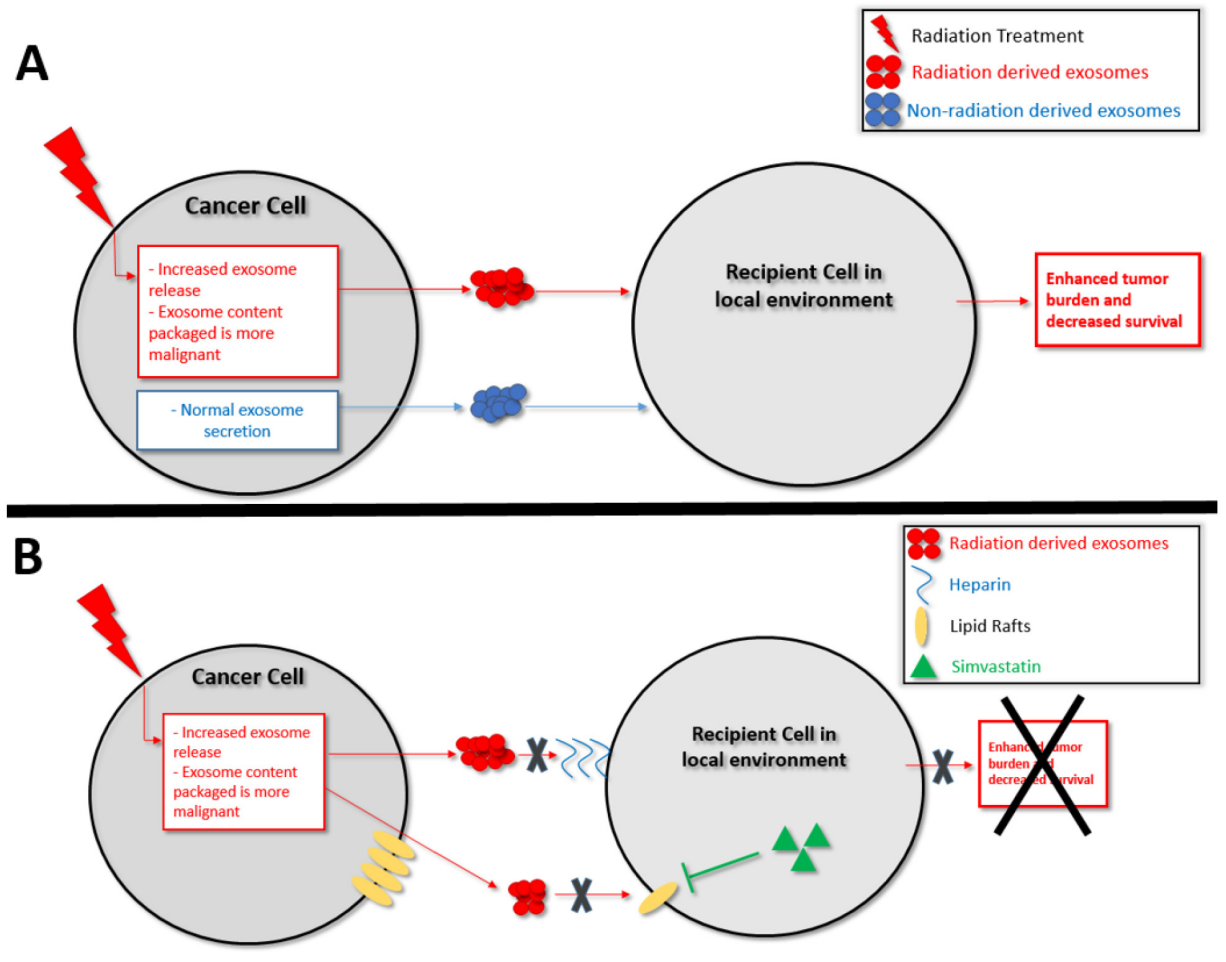
Schematic representation of **(A)** Proposed model for the mechanism of exosomes enhancing the ability of recipient cancer cells to survive radiation therapy **(B)** Proposed model for the therapeutic blockade of exosome uptake.

Based on our findings we propose that one factor underlying a cancer cell's ability to survive radiation may thus be as follows: radiation exposure causes the release of exosomes that have an increase in oncogenic and decrease in tumor-suppressive cargo. Subsequently, neighboring cancer cells internalizing these re-programmed radiation-derived exosomes are activated/disinhibited (Figure 9A). The recipient cell receives more oncogenic RNA (ex. miR-889, Cyclin D1, Annexin A2) and less tumor-suppressive RNA (ex. miR-516, miR-365, TPM1) which then act in that recipient cell to increase its proliferation and ability to survive radiation treatment. The recipient cell also receives more oncogenic proteins involved in the proteasome pathway, Notch pathway, Jak-STAT signaling pathway, and cell cycle pathway. Upregulation of the proteasome pathway has been implicated in glioma aggressiveness and radiation resistance, and proteasome inhibitors are being developed for cancer treatment [74, 75]. STAT3, Notch1/2, Cullin1, TGF-B2, and CREBBP mediate tumor cell proliferation and therapeutic resistance [74–81], and inhibition of the Notch and TGF families sensitizes cancer cells to radiation therapy [79, 82]. The RNA and protein data show that when a cancer cell is stressed with radiation it secretes exosomes with upregulated oncogenic and downregulated tumor-suppressive cargo. This re-programmed cargo is communicated via exosomes and internalized by recipient cells, which subsequently positions the cells to be more apt to survive radiation treatment.

In order for the exosomes to impact the recipient cell, they must be taken up by the cells. DNM2 is critical for exosome uptake, and knockdown of DNM2 decreases exosome internalization [67]. In our study, radiation increased DNM2 in the secreted radiation-derived exosomes. Upregulation of DNM2 is consistent with the increased uptake of radiation-derived exosomes seen when compared to exosomes from non-irradiated cells. The combination of our uptake data and the composition data indicate that a compounding effect occurs that results in the recipient cells not only internalizing increased amounts of radiation-derived exosomes, but these radiation-derived exosomes are also re-programmed to contain and transfer increased oncogenic and decreased tumor-suppressive cargo. Moreover, radiation increased exosome release in a dose-dependent manner. This increase was quantified based upon protein through BCA analysis, as well as with vesicle number based upon nanoparticle tracking analysis. The fold increases in exosome secretion due to radiation were similar in both assays: 3Gy (two-fold increase) and 12Gy (three-fold increase), suggesting that quantification of exosomes through BCA and NTA can be similar.

In a further effort to understand exosome uptake mechanisms we hypothesized that treatment with heparin or simvastatin would block uptake of exosomes. Recipient cells internalize exosomes by a variety of mechanisms. One is mediated through proteoglycan proteins, similar to that of virus particles into recipient cells [34]. This recipient cell-exosome interaction may be inhibited by heparin, a proteoglycan substituted with glycosaminoglycans [34]. Lipid raft mediated internalization is also believed to be an important method of exosome uptake [37]. The use of statins to decrease cellular production of cholesterol and lipids to decrease exosome uptake is being investigated [37]. Simvastatin has the ability to traverse the blood brain barrier and thus is of interest for cancer in the brain [43]. Heparin and simvastatin were effective in inhibiting the effects of radiation-derived exosomes in recipient cells *in vitro* in cell culture and *in vivo* in a murine model of glioblastoma. Although off-target effects of these medications are possible, the fluorescent uptake data in the cell culture model suggest that these treatments are can directly decrease exosome uptake. Simvastatin and the CD81 antibody alone seemed to have minor effects on only the STS26T cells. These effects may be due to the STS26T cell line being more sensitive to therapies as well as the potential for blockade of the endogenously secreted exosomes. Heparin and simvastatin have been shown to decrease cancer cell metastasis and tumor cell proliferation, respectively [39, 41, 83, 84]. Our data suggest that the mechanism behind these effects may be mediated through exosome inhibition. Both Heparin and statins have minimal side effect profiles and these data strongly support further exploration into the use of these agents to minimize radiation resistance in cancer patients. Attempted blockade of exosome uptake with an antibody to the tetraspanin protein CD81 was unsuccessful, corroborating previous studies [32]. The blockage of exosome uptake suggests exosome uptake which is proteoglycan-mediated and lipid raft-mediated may be more critical than tetraspanin-mediated uptake.

In the present study we utilized the U87 glioblastoma, STS26T malignant peripheral nerve sheath tumor, and SH-SY5Y neuroblastoma cell lines. However, *in vivo*, the tumor is highly heterogeneous and includes cancer stem cells (CSCs). When a tumor is treated with radiation, the non-stem cancer cells in the periphery of the tumor mass may be stressed with sublethal doses of radiation causing them to secrete increasingly oncogenic exosomes. Furthermore, the cancer stem cells (CSCs) within the tumor are also exposed to radiation therapy. CSCs are inherently more radioresistant than non-stem cancer cells. Our study suggests that these CSCs may release exosomes that are able to transfer therapeutic resistance functionality to recipient cells. The combination of exosomes secreted by both cancer cells that survive radiation exposure in the margins of the tumor and CSCs may provide recipient tumor cells enhanced cellular proliferation and increased ability to survive radiation, leading to increased tumor burden.

In conclusion, we interrogated three properties of radiation effects on exosomes including exosome function, exosome profile, and exosome uptake/blockade. Our results suggest a novel exosome-based mechanism that may underlie a cancer cell's ability to survive radiation. Furthermore, we elucidate key factors carried by exosomes that may lead to tumor recurrence and subsequent therapeutic resistance. Future studies are warranted to determine how well these findings translate to the clinic and other cancers.

## MATERIALS AND METHODS

### Cell culture, materials, and exosome isolation

SH-SY5Y human neuroblastoma cells, U87 glioma cells from ATCC, and STS26T human malignant peripheral nerve sheath tumor cells, were used. All cell lines were maintained at 37°C in a humidified incubator with 5% CO2. The SH-SY5Y cell line was cultured in Dulbecco's Modified Eagle Medium (DMEM, Life Technologies by Gibco) supplemented with 10% FBS, 1% non-essential amino acids (Gibco), 1% Penicillin-Streptomycin (Gibco), and 200 μg/ml Geneticin (Gibco). The U87 and STS26T cell lines were cultured in Dulbecco's Modified Eagle Medium (DMEM, Gibco) supplemented with 10% fetal bovine serum and 1% Penicillin-Streptomycin (Gibco). Heparin was purchased from Sigma and simvastatin was purchased from Med Chem Express. Before exosome isolation experiments, cell culture media was switched to media supplemented with 10% Exosome-free FBS (System Biosciences). Cells were then immediately either not radiated (control), or radiated at a dosage of 3Gy or 12Gy. The cells were subsequently cultured for 48 hours until 80-90% confluency. Media was aspirated and centrifuged at 3000xG for 15 minutes to purify out cells and cellular debris. The resulting supernatant was incubated with Exo-Quick-TC exosome isolation polymer (System Biosciences) for a minimum of 12 hours at 4°C. The media-ExoQuick combination was centrifuged at 1500xG for 30 minutes. The supernatant was aspirated and the purified exosome pellet was resuspended in 150-300μl dPBS (Gibco).

### Exosome confirmation

Size analysis was performed using the ZetaSizer particle size analyzer (Malvern Instruments) by taking 10 μl of each exosome solution and resuspending in 1mL of dPBS in a cuvette, which was subsequently placed into the instrument and read. Exosomes were quantified using a BCA Assay (Thermo-Scientific) or with nanoparticle tracking analysis (NanoSight NS300). Transmission electron microscopy was performed by taking 10 μl of exosome solution and placing them on parafilm. Formvar coated copper grids were then placed on top of the drops and incubated for 20 minutes. The copper grids were incubated with a 4% solution of paraformaldehyde in 0.1M PBS for 20 minutes, washed thrice with PBS for 1 minute each, incubated with 1% glutaraldehyde in 0.1M PBS for 5 minutes, washed with distilled water for 2 minutes, washed thrice with PBS for 2 minutes each, negatively stained with 1% Uranyl acetate for 20 seconds, and observed by transmission electron microscopy (JEOL-1400).

### Immunoblot analysis

Protein expression was determined with immunoblot analysis. Exosome concentration was determined using BCA assay (Thermo-Scientific). 5μg of exosomes was solubilized on nitrocellulose membrane. The membrane was then blocked with 5% milk in TBS-T for 1 hour. Membranes were incubated overnight at 4°C with primary antibodies for CD81 (1:200 sc-166029), CD63 (1:200 Ab134045), tsg101 (1:200 sc-7964. The membrane was washed thrice with 1X TBS-T. Secondary antibodies were incubated for 1 hour and the membranes were again washed thrice with 1X TBS-T. The blots were then subsequently imaged with GE Amersham Imager 600.

### Cellular proliferation analysis

4 × 10^5^ SH-SY5Y cells, 4 × 10^3^ U87 cells, and 4 × 10^3^ STS26T cells were plated on 96 well plates and allowed to adhere overnight. The following day, these cells were incubated with PBS control, non-radiation derived exosomes, or radiation derived exosomes individually, in quadruplicate. These cells were allowed to proliferate for 48 hours and analysis of cell proliferation was performed using an MTS-PMS Assay (Promega) according to manufacturer's protocol. Naïve cells were incubated with exosomes at a concentration of 30μg/mL. Data is expressed as a ratio of naïve cells exposed to control.

### Apoptosis assay

4 × 10^5^ wild type SH-SY5Y cells, 4 × 10^3^ U87 cells, and 4 × 10^3^ STS26T cells were plated on 96 well plates and allowed to adhere overnight. The following day, these cells were incubated with PBS control, non-radiation derived exosomes, or radiation derived exosomes individually, in quadruplicate. After a 24 hour incubation, 96 well plates were radiated. 24 hours after radiation, analysis of cell death was performed using an MTS-PMS Assay (Promega) according to manufacturer's protocol. Naïve cells were incubated with exosomes at a concentration of 30μg/mL. Radiation dosages of 3Gy and 12Gy were used. Data is expressed as a ratio of naïve cells exposed to control.

### Reactive oxygen species assay

25 × 10^3^ U87 cells and 25 × 10^3^ STS26T cells were plated on 96 well plates and allowed to adhere overnight. The following day, these cells were incubated with PBS control, non-radiation derived exosomes, or radiation derived exosomes individually, in quadruplicate. Exosomes were treated at a concentration of 30ug/mL. After a 24 hour incubation, media was removed and two 5 minute PBS washes were performed. PBS was removed and analysis of reactive oxygen species was performed using a H2DCFDA assay (Promega) according to manufacturer's protocol. Briefly, 3mg of H2DCFDA was solubilized in 300uL DMSO, which was subsequently mixed with 20mL of PBS. 100uL of the H2DCFDA solution was added to wells. 96 well plates were radiated. Plates were incubated for 30 minutes to 3 hours and read on a plate reader (Gemini EM) at 492-495nm excitation and 517-527nm emission. Data is expressed as a ratio of naïve cells exposed to control.

### Exosome blockade analysis

2 × 10^3^ U87 cells and 2 × 10^3^ STS26T cells were plated on 96 well plates and allowed to adhere overnight. The following day, one set of each type of cells was incubated with simvastatin (2uM)[37]. Simvastatin was chosen for its blood brain barrier permeability making it relevant for gliomas [85]. 24 hours after incubation, another set of cells was incubated with heparin (20ug/mL) for 30 minutes [34, 36, 86]. The radiation derived exosomes were aliquoted and one aliquot was incubated with heparin (20ug/mL) for 30 minutes at room temperature and another exosome aliquot was incubated with anti-CD81 antibodies (20ug/mL) (sc-166029) for 30 minutes at room temperature [32]. The cells were incubated with PBS control, non-radiation derived exosomes, radiation derived exosomes, radiation derived exosomes plus heparin, radiation derived exosomes plus simvastatin, radiation derived exosomes plus anti-CD81 antibodies, anti-CD81 antibody alone, heparin alone, or simvastatin alone, individually, in quadruplicate. Exosomes were treated at a concentration of 30ug/mL. Cellular proliferation analysis and cell survival analysis was performed 24 hours later as previously described in *2.4* and *2.5*, respectively.

### Exosome fluorescent tagging and blockade analysis

Exosomes were isolated as described previously in *2.1*. The exosomes were fluorescently labeled with PKH67 following manufacture's protocol. Briefly, exosome pellets were resuspended in 1 mL Diluent C. Separately, 1 mL Diluent C was mixed with 4 μL PKH67. The Diluent C-PKH67 solution was incubated with the exosomes for four minutes. The fluorescent labeling reaction was stopped by adding an equal volume of 1% BSA. Labeled exosomes were ultracentrifuged at 110,000 × G for 60 minutes, washed with PBS, and ultracentrifuged again at 110,000 × G for 60 minutes.

5 × 10^3^ U87 cells and 5 × 10^3^ STS26T cells were plated in 8 well chamber slides and allowed to adhere overnight. The following day, simvastatin (2uM) was added as described in *2.7* and allowed to incubate for 24 hours. After 24 hours, heparin (20ug/mL) or anti-CD81 antibodies (20ug/mL) were added to cells and exosome aliquots and incubated for 30 minutes, as described in *2.7*. The cells were incubated with PBS control, non-radiation derived exosomes, radiation derived exosomes, radiation derived exosomes plus heparin, radiation derived exosomes plus simvastatin, or radiation derived exosomes plus anti-CD81 antibodies. Exosomes were treated at a concentration of 10ug/mL. 24 hours after incubation, media was removed and wells were washed twice with PBS for 5 minutes each. The wells were incubated with DAPI (1:1000) for 10 minutes. The wells were washed twice with PBS for 5 minutes each wash. Subsequently, the wells were fixed with 10% formaldehyde for 20 minutes and again washed twice with PBS for 5 minutes each wash. A drop of gel mount media was placed on the slides and they were mounted with coverslips and allowed to dry in the dark overnight. Slides were viewed under a fluorescence microscope (Nikon Eclipse 80i).

### *In vivo* studies

Seven groups of nude mice were injected with 1 × 10^6^ U87-Luciferase cells subcutaneously in the mouse flank. This well-established glioma cell line transfected with luciferase allows for accurate *in vivo* tumor quantification. One week after injection of tumor cells, the mice were imaged using Intravital Imaging Spectroscopy (IVIS). A linear relationship between the bioluminescent intensity and the tumor weight is evident by earlier reports [87] and is also able to accurately monitor tumor progression over time [88, 89]. The mice were first anesthetized within an induction chamber using a concentration of 5% isoflurane. Next, a subcutaneous injection of 100μL of luciferin-D Substrate (purchased from Caliper LS and diluted in 35 mL of dH2O with a final concentration of 28.57 mg/mL) was administered. These mice were weighed and then transferred into the imaging chamber where anesthesia was maintained with a concentration of 1-2% isoflurane emitted through nose cones. Five minutes post-injection of luciferin-D, imaging utilizing the IVIS 50 (Perkin-Elmer) was performed according to manufacturer's protocol (Perkin Elmer). The IVIS was run for 0.5 seconds and bioluminescence was recorded. Once it was confirmed that there was a measurable signal from the tumor cells, the intensity of bioluminescence was measured utilizing *LivingImage* software. All values were ranked in order or bioluminescent signal intensity and then normalized and evenly distributed into seven homogeneous groups. Mice whose tumor spontaneously regressed were removed from the study. Groups consisted of mice treated with weekly intra-tumoral injections of either saline control (n=6), non-radiation derived exosomes (n=7), radiation derived exosomes (n=6), radiation derived exosomes plus subcutaneous heparin (n=6), radiation derived exosomes plus oral simvastatin (n=6), heparin alone (n=6), or simvastatin alone (n=6). 50ug of exosomes in PBS were given [90]. Heparin was solubilized in PBS and given daily subcutaneously at a concentration consistent with the current prophylactic dose in humans, 100IU/kg [91]. Simvastatin was solubilized in Ora-Plus (Perrigo) and given daily as an oral gavage at a concentration of 10mg/kg. This dosage was shown previously to not affect tumor progression in mice [84] and was chosen to isolate the exosome blockade effect. Mice were treated with 3Gy radiation weekly by covering their bodies except for the site of the tumor with lead. Tumor growth was analyzed through IVIS imaging and quantification weekly. Mouse survival was assessed through end point analysis which was defined as mouse death from tumor by cachexia or the veterinarians deeming the tumor to be large enough that necessitated the veterinarian to perform mouse sacrifice.

### Immunohistochemistry of tumor tissue sections

In order analyze expression of tumor proliferation and apoptosis proteins after tumor treatment, tumor tissues were obtained from mice in each group and H&E and immunohistochemistry was performed. The tumor tissues were frozen and sectioned to 10μm using a cryostat and subjected to immunohistochemistry to identify protein expression. The sections were fixed with 4% paraformaldehyde for 20 minutes and washed with PBS for 5 minutes. The non-specific binding sites on the tissues were blocked with 10% normal goat serum for 1 hour at room temperature. Subsequently, the tumor tissues were incubated with Ki67 antibody (1:500 ab15580, Abcam) or Cleaved Caspase 3 antibody (1:500 9661S, Cell Signaling) overnight at 4°C and washed with phosphate buffered saline (PBS) three times for 5 minutes each wash. The sections were treated with DAPI (1:1000) and secondary Alexa Fluor 488 conjugated anti-rabbit IgG (1:200) for 60 minutes and washed with PBS 3 times for 5 minutes before gel mounting, drying overnight, and viewing under a fluorescence microscope (Nikon Eclipse 80i).

### RNA analysis

Exosomes were isolated as previously described in *4.*1. Briefly, cells flasks were either not radiated, radiated at 3Gy, or radiated at 12Gy. Exosomes from flasks were isolated and run for RNA exploration analysis. Total exosomal RNA was extracted using mirVana kit (Ambion, cat#:AM1560). The extracted RNAs were quantified and quality checked using a BioAnalyzer RNA 6000 Pico Kit (Agilent Technologies). QuantSeq 3′ mRNA-Seq Library Prep Kit FWD for Illumina (Lexogen) was used to generate mRNA-seq libraries as per manufacturer's recommendation, followed by deep sequencing on an Illumina HiSeq 2500 as per the manufacturer's instructions. Briefly, 0.5-1 ng of total RNA was subjected to the first cDNA strand which is initiated by oligodT priming. The synthesis of the second cDNA strand is performed by random priming, in a manner that DNA polymerase is efficiently stopped when reaching the next hybridized random primer, so only the fragment closest to the 3′ end gets captured for later indexed adapter ligation and PCR amplification. The processed libraries were assessed for fragment size distribution and quantity using a BioAnalyzer High Sensitivity DNA kit (Agilent Technologies). Pooled libraries were denatured and loaded onto a TruSeq Rapid flow cell on an Illumina HiSeq 2500 and run for 50 cycles using a single-read recipe according to the manufacturer's instructions. De-multiplexed sequencing reads passed the default purify filtering of Illumina CASAVA pipeline (released version 1.8) were subjected to QuantSeq data analysis pipeline on a Bluebee genomics analysis platform (Bluebee). Small RNA-seq libraries was generated by NEXTflex Small RNA Library Prep Kit v3 for Illumina (BioO Scientific), followed by deep sequencing on an Illumina HiSeq 2500 as per the manufacturer's instructions. Briefly, 1-2 ng of total RNA was ligated with chemically modified 3′- and 5′- adapters that can specifically bind to mature micro RNAs, followed by reverse transcription and PCR amplification. Unique index sequence tags were introduced during PCR to enable multiplexed sequencing. Each library was assessed for the presence of desired micro RNA population and approximate library quantity by Bioanalyzer High Sensitivity DNA Kit (Agilent Technologies). Pooled libraries were denatured and loaded onto a TruSeq Rapid flow cell on an Illumina HiSeq 2500 and run for 50 cycles using a single-read recipe according to the manufacturer's instructions. De-multiplexed sequencing reads passed the default purify filtering of Illumina CASAVA pipeline (released version 1.8) were quality trimmed/filtered using The FASTX-Toolkit (http://hannonlab.cshl.edu/fastx_toolkit). The filtered reads were further trimmed with both 5′ and 3′ adapter sequences and subjected to Chimira suite to align and count miRNA expression [92]. For both mRNA and small RNA-seq datasets, TCC v1.14.0 R package [93] was used to identify differentially expressed genes (DEG) between the non-radiated (0Gy) and radiated (3Gy and 12Gy) exosomal RNA read counts. We used edgeR as a test method [94]. Significantly DEX between control and radiated samples (n=2) were defined to be those with q-value < 0.1. We used Ingenuity Pathway Analysis (IPA) to identify overrepresented mRNA in the pathways and their effects in various functional contexts, such as subcellular location, functional gene family, association with drugs, pathways, and disease relevance.

### Proteomic analysis

Exosomes were isolated as previously described in *4.*1. Briefly, cells flasks were either not radiated, radiated at 3Gy, or radiated at 12Gy. Exosomes from flasks were isolated and run for protein exploration analysis. Exosomal protein from the samples were analyzed utilizing the Tandem Mass Tag (TMT)-10plex kit according to manufacturer's protocol (Thermofischer Scientific). This kit provides multiplexed protein identification and quantitative analysis by tandem mass spectrometry. Briefly, exosomal proteins were normalized to 25ug of exosome proteins in 40uL of PBS for each of the groups (non-radiation derived exosomes, 3Gy radiation derived exosomes, and 12Gy radiation derived exosomes) (n=3). The exosomal proteins were incubated with lysis buffer and then centrifuged at 16,000 × G for 10 minutes. The supernatant was then mixed with 100mM TEAB, 200mM TCEP, for one hour, and subsequently 5μL of the 375mM iodoacetamide for 30 minutes. Acetone was then added and allowed to precipitate overnight. The samples were centrifuged at 8000 × G for 10 minutes and the pellets were then resuspended in 100mM TEAB. Trypsin storage solution and trypsin were added to the samples and digested overnight at 37°C. These samples were then labeled with the TMT label reagents with 1 hour incubation at which point 5% hydroxylamine was then added to quench the reaction. The samples were then run on the Orbitrap Velos Mass Spectrometer (Thermofischer Scientific) and the data was subsequently processed using Proteome Discoverer (version 2.1). The search engine is SequestHT. PSMs are filtered using Percolator. Proteome Discoverer uses its own algorithm for protein grouping. Samples were searched against human database and filtered to retain proteins/peptides with <1% FDR. Adavita iPathwayGuide (Advaita Bioinformatics) Next-gen pathway analysis software with the Impact Analysis method was then used to identify dysregulated protein relationships and their effects in various functional contexts, such as subcellular location, gene ontology, association with drugs, pathways, and disease relevance.

### Statistical analysis

All of the data generated in the proposed experiments were subjected to statistical analysis. GraphPad Prism 4.03 (GraphPad Software, San Diego, CA) was used for statistical analysis and groups were analyzed using one way ANOVA with Tukey-Kramer posttest unless otherwise noted in previous methods. Survival analysis was performed using Kaplan-Meier curves and curves were compared by means of a log rank test. At least three replicates were performed unless otherwise noted in previous methods. A p value <0.05 was deemed significant.

## Abbreviations

CD81: Cluster of differentiation 81
CD63: Cluster of differentiation 63
TSG101: Tumor susceptibility 101
ROS: Reactive oxygen species
Gy: Gray
H & E: Hematoxylin and Eosin
NPM1: Nucleophosmin 1
ACTG1: Actin Gamma 1
VAMP8: Vesicle Associated Membrane Protein 8
RL15: Ribosomal Protein L15
FUT11: Fucosyltransferase 11
ZFR: Zinc Finger RNA Binding Protein
CCND1: Cyclin D1
ANXA2: Annexin A2
SCD: Stearoyl-CoA desaturase
DNM2: Dynamin 2
DERL1: Derlin 1
CISD1: mitoNEET
WWC1: Kibra
PPIC: Peptidylprolyl Isomerase C
TPM1: Tropomyosin 1
LRRFIP1: LRR Binding FLII Interacting Protein 1
TSPAN5: Tetraspanin 5
STAT4: Signal Transducer And Activator Of Transcription 4
CGGBP1: CGG Triplet Repeat Binding Protein 1
TGF-B2: Transforming Growth Factor-Beta 2
CREBBP: cAMP-response element binding protein
